# Osseodensification Versus Subtractive Drilling in Cortical Bone: An Evaluation of Implant Surface Characteristics and Their Effects on Osseointegration

**DOI:** 10.3390/biomimetics10100662

**Published:** 2025-10-01

**Authors:** Sara E. Munkwitz, Albert Ting, Hana Shah, Nicholas J. Iglesias, Vasudev Vivekanand Nayak, Arthur Castellano, Lukasz Witek, Paulo G. Coelho

**Affiliations:** 1 University of Miami Miller School of Medicine, Miami, FL 33136, USA; 2 Florida International University Herbert Wertheim College of Medicine, Miami, FL 33199, USA; 3 DeWitt Daughtry Family Department of Surgery, University of Miami Miller School of Medicine, Miami, FL 33136, USA; 4 Department of Biochemistry and Molecular Biology, University of Miami Miller School of Medicine, Miami, FL 33136, USA; 5 Dr. John T. Macdonald Foundation Biomedical Nanotechnology Institute (BioNIUM), University of Miami, Miami, FL 33136, USA; 6 Mackenzie Evangelical School of Medicine Paraná, Curitiba 80730-000, PR, Brazil; 7 Federal University of Paraná, Curitiba 80060-000, PR, Brazil; 8 Biomaterials and Regenerative Biology Division, NYU College of Dentistry, New York, NY 10010, USA; 9 Department of Biomedical Engineering, NYU Tandon School of Engineering, Brooklyn, NY 11201, USA; 10 Hansjörg Wyss Department of Plastic Surgery, NYU Grossman School of Medicine, New York, NY 10016, USA; 11 Department of Oral and Maxillofacial Surgery, NYU College of Dentistry, New York, NY 10010, USA; 12 DeWitt Daughtry Family Department of Surgery, Division of Plastic Surgery, University of Miami Miller School of Medicine, Miami, FL 33136, USA; 13 Sylvester Comprehensive Cancer Center, University of Miami Miller School of Medicine, Miami, FL 33136, USA

**Keywords:** osseodensification, cortical bone, acid etching

## Abstract

Osseodensification (OD) has emerged as a favorable osteotomy preparation technique that preserves and compacts autogenous bone along the osteotomy walls during site preparation, enhancing primary stability and implant osseointegration. While OD has demonstrated promising results in low-density trabecular bone, especially when used in conjunction with acid-etched (AE) implant surfaces, its efficacy in high-density cortical bone remains unclear—particularly in the context of varying implant surface characteristics. In this study, Grade V titanium alloy implants (Ti-6Al-4V, 4 mm × 10 mm) with deep threads, designated bone chambers and either as-machined (Mach) or AE surfaces were placed in 3.8 mm diameter osteotomies in the submandibular region of 16 adult sheep using either OD or conventional (Reg) drilling protocols. Insertion torque values (N·cm) were measured at the time of implant placement to evaluate primary stability. Mandibles were harvested at 3-, 6-, 12-, or 24-weeks post-implantation (*n* = 4 sheep/time point), and histologic sections were analyzed to quantify bone-to-implant contact (BIC) and bone area fractional occupancy (BAFO). Qualitative histological analysis confirmed successful osseointegration among all groups at each of the healing time points. No statistically significant differences were observed between OD and conventional drilling techniques in insertion torque (*p* > 0.628), BIC (*p* > 0.135), or BAFO (*p* > 0.060) values, regardless of implant surface type or healing interval. The findings indicate that neither drilling technique nor implant surface treatment significantly influences osseointegration in high density cortical bone. Furthermore, as the osteotomy was not considerably undersized, the use of OD instrumentation showed no signs of necrosis, inflammation, microfractures, or impaired osseointegration in dense cortical bone. Both OD and Reg techniques appear to be suitable for implant placement in dense bone, allowing flexibility based on surgeon preference and clinical circumstances.

## 1. Introduction

Primary implant stability is a key biomechanical factor influencing successful osseointegration and long-term clinical outcomes in both dental and orthopedic applications [1]. Successful mechanical anchorage relies on both primary implant stability and the initiation of cellular processes that lead to secondary biologic stability [2,3,4]. Osseointegration can therefore be defined as the endpoint of these mechanical and biological interactions with surrounding bone, providing a direct structural and functional connection. This process is influenced by multiple factors, including bone density [5], implant surface characteristics [6,7], and drilling protocols [6]. Among these, the method of osteotomy preparation plays a critical role in determining the quality of the bone–implant interface and the resultant mechanical stability [8]. Conventional implant site preparation involves subtractive drilling (Reg) using sequential cutting burs to remove bone and create the desired osteotomy dimensions. This may reduce the volume of native bone available for implant engagement, and consequently, this approach has been associated with lower insertion torque values and decreased bone-to-implant contact (BIC) [9].

To address the limitations associated with subtractive drilling, osseodensification (OD) has been introduced as a promising alternative for osteotomy preparation [9]. OD utilizes specially designed burs featuring negative rake angles with documented use in both clockwise (CW) and counterclockwise (CCW) directions [10]. Rather than excising bone, the non-cutting compaction burs condense autogenous bone chips laterally and apically along the osteotomy wall, thereby increasing local bone density around the implant site [11]. This dual functionality may be particularly valuable in anatomical areas with heterogenous bone density, as it allows for tailored osteotomy preparation based on bone quality. OD has demonstrated efficacy in low-density bone, which is characterized by a porous, trabecular architecture with reduced mineral content and low mechanical stiffness [9,12,13]. This bone type is commonly found in anatomical regions such as the maxilla, posterior mandible, condyle, iliac crest, and epiphyses of long bones, where it serves as a scaffold for marrow space and facilitates metabolic exchange [14,15]. In this bone type, the compaction effect achieved through OD has been shown to improve primary implant stability [11]. Importantly, the autologous bone fragments preserved during the OD process have been shown to serve as nucleation sites for new bone formation, contributing to increased bone-to-implant contact (BIC) and higher bone area fractional occupancy (BAFO) compared to conventional drilling techniques [11].

In contrast to trabecular bone, high-density cortical bone presents a different biomechanical environment. Characterized by high mineral content, structural rigidity, and minimal intrinsic porosity, cortical bone is predominantly located in load-bearing regions such as the diaphyseal areas of long bones, the anterior mandible [16,17]. While the benefits of OD in trabecular bone are well documented, current evidence regarding its efficacy in cortical bone remains inconclusive, with studies reporting mixed outcomes [18,19,20,21]. For example, a human cadaveric mandible study by Mercier et al. demonstrated that OD significantly enhanced implant stability and insertion torque compared to conventional drilling techniques [18]. Similarly, Vaddamanu et al. conducted a comparative study using pre- and post-operative computed tomography imaging of human mandibles and maxillae, reporting a significant increase in peri-implant bone density at apical, mesial, and distal sites following OD [19]. Conversely, an ex vivo study using dense bovine femur bone found no significant improvement in implant stability with OD relative to conventional methods, suggesting that the high intrinsic density of cortical bone may limit the perceived compaction benefits associated with OD [20]. Moreover, a recent randomized clinical trial reported comparable outcomes between OD and conventional subtractive drilling in sites with high-density bone, as evidenced by similar insertion torque values and implant stability measurements [21].

In addition to drilling protocols, implant surface characteristics play a critical role in promoting osseointegration [22]. Acid etching (AE) is a widely employed surface modification technique that enhances implant surface area by generating microscale topographical features through controlled chemical corrosion [21]. This process not only removes potential surface contaminants but also increases surface roughness and surface energy, thereby improving wettability, cell adhesion, and activation [23,24,25,26,27]. AE-treated surfaces have been shown to enhance osteoblastic activity and promote new bone formation, particularly in trabecular bone environments [28,29,30]. The combination of OD and AE in trabecular bone has demonstrated a synergistic effect, enhancing both primary mechanical stability and secondary biological integration of the endosteal implants [31]. In contrast, while several studies have evaluated AE in conjunction with other surface treatments such as grit blasting and reported favorable outcomes in cortical bone [29,30,32,33,34,35], the isolated effects of AE in dense cortical bone remain underexplored. Additionally, there is a dearth of studies that specifically investigate the potential combinatory effects of employing OD and AE in high-density cortical bone. Therefore, the present study aimed to evaluate the impact of OD on osseointegration outcomes in cortical bone and to assess whether implant surface characteristics modulate these effects.

## 2. Materials and Methods

### 2.1. Materials

**Figure 1:**
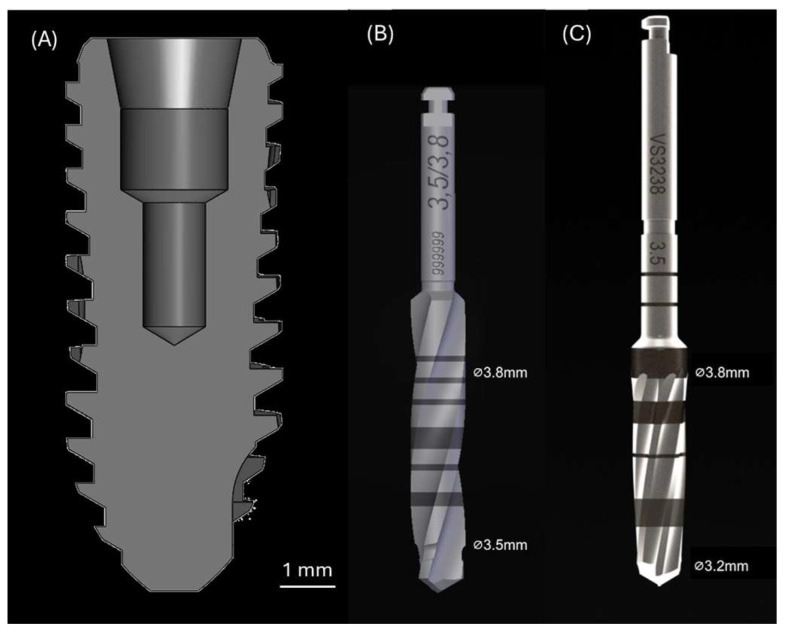
(**A**) Computer-aided design of the implant used in the current study. Image courtesy: Versah LLC, Jackson, MI, USA. Pictographs of the (**B**) Reg drill and (**C**) OD drill. Reprinted from [36], with permission from Elsevier.

Custom-manufactured conical Grade V titanium alloy (Ti-6Al-4V) implants with deep threads and designated bone chambers (Versah LLC, Jackson, MI, USA; shown in Figure 1A), measuring 4 mm in diameter and 10 mm in length, were utilized in this study to be placed in 3.8 mm diameter osteotomies. The implant size was chosen to ensure that the osteotomy diameter closely matched the implant’s major diameter to avoid undersizing. Two surface conditions were evaluated: (1) machined (Mach) surface—as received from the manufacturer; and (2) acid-etched (AE) surface—altered through a controlled chemical etching process to modify the surface topography. To generate the AE surface, Mach implants were subjected to acid etching using 37% hydrochloric acid. The implants were equally distributed across both surface conditions (Mach or AE), as well as drilling techniques (Reg or OD). All implants were sterilized by gamma irradiation prior to in vivo experimentation.

### 2.2. Preclinical In Vivo Model

The in vivo study was conducted following approval from the Institutional Animal Care and Use Committee of École Nationale Vétérinaire d’Alfort (Maisons-Alfort, Ile-de-France, France; file and notice numbers: 13-011 and 05/14/13-3, respectively). Sheep were selected as the animal model to facilitate translational relevance. The mandible was chosen as the implantation site due to its size and cortical bone architecture. A total of 16 adult sheep, weighing ~55 kg, were acquired and allowed to acclimate for ~1 week at the animal facility prior to any surgical intervention. The sheep were group-housed, received daily enrichment, and had unrestricted access to food and water. All surgical procedures were performed under general anesthesia in a sterile environment. The animals underwent overnight fasting before surgery. Anesthesia was induced intravenously with sodium pentothal (15–20 mg/kg) in Normasol solution into the jugular vein and maintained with inhaled isoflurane (1.5–3%) in O_2_/N_2_O (50/50). Physiological monitoring included ECG, waveform capnography, SpO_2_, and body temperature. Thermal regulation was maintained using a circulating warm water blanket. Prior to surgery, the operative sites were shaved and disinfected using an iodine-based antiseptic solution. An incision was made along the submandibular region, and soft tissues were carefully retracted to expose the underlying mandibular bone. Two distinct osteotomy techniques were used: (1) Reg—performed in a three-step sequence consisting of a 2.0 mm pilot, followed by sequential enlargement using universal twist drills with 3.2 mm and 3.8 mm coronal diameters (Figure 1B); and (2) OD—performed in a counterclockwise direction using Densah Burs (Versah LLC, Jackson, MI, USA), also in a 3-step sequence using a 2.0 mm pilot, followed by 2.8 mm (coronal diameter; VS2228) and 3.8 mm (coronal diameter; VS3238) multi-fluted tapered burs (Figure 1C). All osteotomy preparations were performed at 1100 rpm under continuous saline irrigation. Each animal received 4 implants (2 per mandibular side), resulting in a total of 64 implants (*n* = 16/experimental group). Experimental conditions were randomized and interpolated within each animal to minimize anatomical site bias. Insertion torque (N·cm) was recorded at the time of placement using a digital torque meter (STC2-G, Tonichi Mfg. Co. Ltd., Tokyo, Japan). Subsequently, the surgical site was closed using Vicryl 2-0 for fascial closure and 2-0 nylon for skin closure. Cefazolin (500 mg) was intravenously administered before and after surgery. Post-operatively, food and water were offered to the animals ad libitum. Analgesics (transdermal fentanyl, 3 μg/h/kg) and anti-inflammatory medications (meloxicam 0.5 mg/kg, IM) were administered for 3 and 2 days post-operatively, respectively. Euthanasia was performed at 3-, 6-, 12-, or 24-weeks postoperatively (*n* = 4 sheep/time point) following the approved protocol. Following euthanasia, the mandibles were harvested by sharp dissection. Samples exhibiting clinical signs of infection and/or implant mobility were excluded from analysis.

### 2.3. Histologic Procedures and Histomorphometric Analysis

Non-decalcified histological processing was performed on the implant-containing mandibular bone samples. Samples were initially fixed in 10% buffered formalin for 24 h, followed by ethanol dehydration (70–100%). The samples were subsequently infiltrated and embedded in methacrylate-based resin, then sectioned (~300 μm thick) using a low-speed diamond precision saw (Isomet 2000, Buehler Ltd., Lake Bluff, IL, USA), with cuts aligned with the longitudinal axis of the implant. Sections were glued to acrylic slides using a low-viscosity cyanoacrylate adhesive (Loctite 408, Henkel AG & Co. KgaA, Dusseldorf, Germany). After 24 h setting time, the mounted specimens were ground under continuous irrigation using silicon carbide abrasive papers of progressively finer grit sizes (400, 600, 800, and 1200) to a final thickness of ~90 μm. The slides were subsequently polished with a microfiber cloth and an alumina suspension (1 µm MicroPolish^TM^ Alumina Suspension Buehler Ltd., Lake Bluff, IL, USA). To visualize bone and soft tissue, the slides were stained with Stevenel’s Blue and Van Gieson’s Picro Fuchsin (SVG). High-resolution digital images were acquired using an automated slide scanner (CS2 and Imagescope v12.4.6, Leica Biosystems, Deer Park, IL, USA). Histomorphometric evaluation was carried out on 1 histological section per implant using image analysis software (ImageJ v1.51, NIH, Bethesda, MD, USA) to assess bone-to-implant contact (BIC) (Equation (1)), and bone area fractional occupancy (BAFO) (Equation (2)).

(1) $$BIC\left(\%\right)=\frac{Length\;of\;bone\;in\;direct\;contact\;with\;surface\;of\;healing\;chambers}{Total\;length\;of\;all\;healing\;chambers}\times 100\%$$

(2) $$BAFO\left(\%\right)=\frac{Area\;of\;bone\;within\;healing\;chambers}{Total\;area\;of\;all\;healing\;chambers}\times 100\%$$

### 2.4. Statistical Analysis

Statistical analysis was performed using SPSS (v29, IBM Corp., Armonk, NY, USA). Mechanical testing data (insertion torque) and histomorphometric parameters (BIC and BAFO) are presented as mean values with the corresponding 95% confidence intervals (mean ± 95% CI), unless otherwise specified. Data were analyzed using mixed models with fixed effects including implant surface treatment (Mach or AE), drilling technique (Reg or OD), and time in vivo (3, 6, 12, or 24 weeks). Statistical significance was set at *p* < 0.05.

## 3. Results

**Figure 2:**
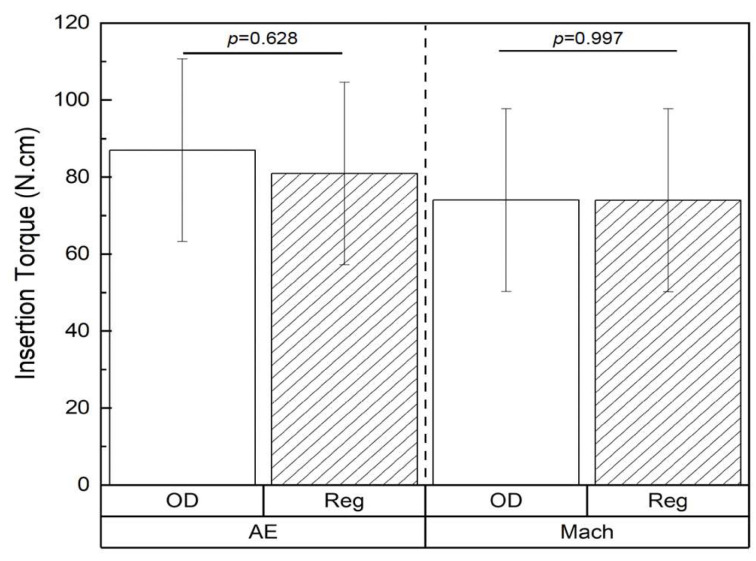
Insertion torque (N·cm) values as a function of surface treatment and drilling technique. Data presented as mean ± 95% CI. *p* < 0.05 is statistically significant.

The insertion torque values were statistically similar between the OD and Reg drilling techniques irrespective of the surface characteristics of the implants (AE: 87.00 ± 21.4 N·cm (OD) vs. 80.95 ± 21.4 N·cm (Reg), respectively, *p* = 0.689; and Mach: 74.04 ± 21.4 N·cm (OD) vs. 74.00 ± 21.4 N·cm (Reg), respectively, *p* = 0.997) (Figure 2).

**Figure 3:**
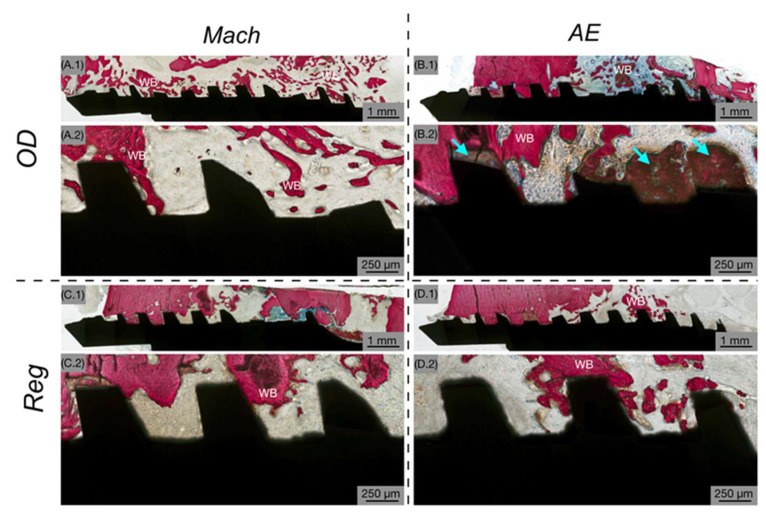
Representative histomicrographs at 3 weeks showing Mach (**A**,**C**) and AE (**B**,**D**) implants with OD (**A**,**B**) and Reg (**C**,**D**) drilling techniques. The implant is shown in black, while the bone is shown in red. Low magnification (**A.1**–**D.1**) and high magnification (**A.2**–**D.2**) histological images show predominantly loose connective tissue surrounding implants, with the presence of woven bone (WB). Bone chips (blue arrows) are shown as a result of the OD drilling technique.

Gross examination of samples following euthanasia revealed no evidence of infection, inflammation or adverse tissue reactions; therefore, no samples were excluded from the analysis. The evaluation of histological micrographs confirmed successful osseointegration in all implant groups at each healing interval. Independent of surface treatment or osteotomy technique, bone tissue was consistently observed in direct contact with the implant surfaces, accompanied by histological evidence of ongoing bone remodeling throughout the healing process. At 3 weeks post-implantation, newly formed woven bone was observed surrounding the implant threads and occupying the healing chambers, indicating an active osteogenic phase (Figure 3). At low magnification (Figure 3(A.1–D.1)), predominantly loose connective tissue was seen surrounding the implants, with minimal woven bone. At higher magnification (Figure 3(A.2–D.2)), the bone was infrequently in close contact with the implant’s surface and hardly within the healing chambers of the threads.

**Figure 4:**
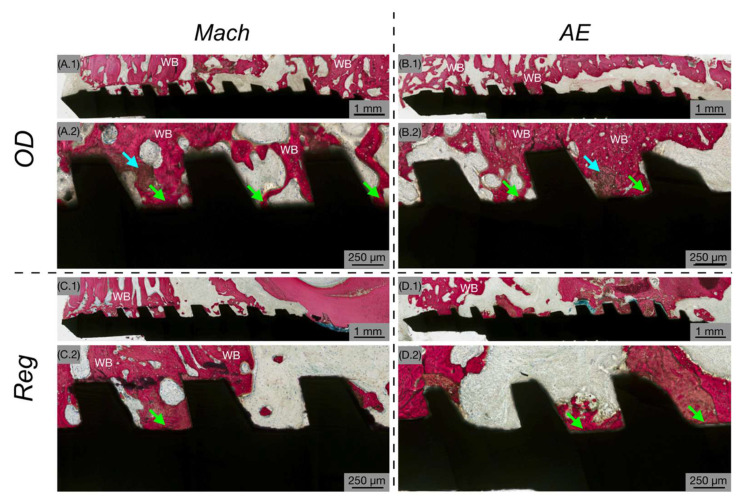
Histological analysis at 6 weeks showing Mach (**A**,**C**) and AE (**B**,**D**) implants with OD (**A**,**B**) and Reg (**C**,**D**) drilling techniques. The implant is shown in black, while bone is shown in red. Low magnification (**A.1**–**D.1**) demonstrates increased bone fill within healing chambers and a more continuous bone presence around the implant. High magnification (**A.2**–**D.2**) shows an increase in bone that is in direct contact with the implant surface (green arrows) and several bone chips (blue arrows) as a result of the OD drilling technique.

By 6 weeks post-implantation, increased woven bone formation was evident within the healing chambers in direct contact with the implant surface (Figure 4). Low-magnification images (Figure 4(A.1–D.1)) showed increased bone fill within the healing chambers and a more continuous presence of bone surrounding the implants. High-magnification views (Figure 4(A.2–D.2)) demonstrated an increase in bone directly contacting the implant surface indicating ongoing osseointegration. In the OD-prepared osteotomies, remaining bone chips served as nucleation sites for new bone formation, irrespective of the implant’s surface characteristics (Figure 4(A.2,B.2)).

**Figure 5:**
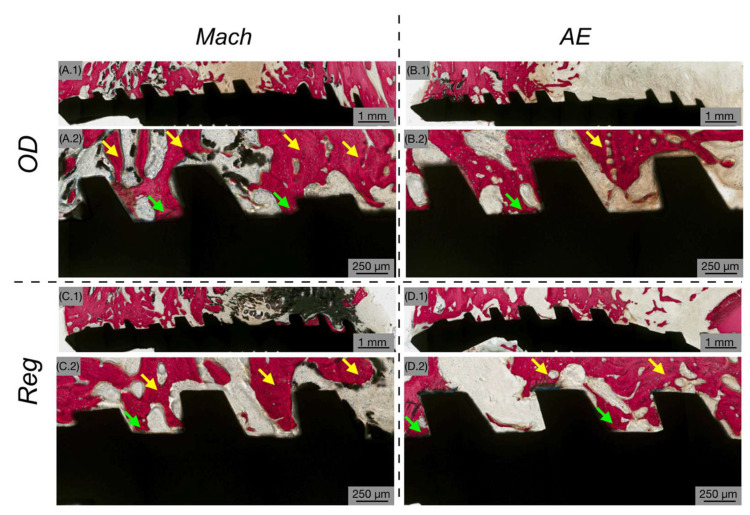
Histological analysis at 12 weeks showing Mach (**A**,**C**) and AE (**B**,**D**) implants with OD (**A**,**B**) and Reg (**C**,**D**) drilling techniques. The implant is shown in black, while bone is shown in red. At low magnification (**A.1**–**D.1**), more mature hard tissue architecture is evident, with bone occupying the healing chambers and surrounding the implant. High magnification (**A.2**–**D.2**) confirms the predominance of bone remodeling sites (yellow arrows). The green arrows highlight the bone that is in close contact with the implant surface.

At 12 weeks in vivo, there was a noticeable shift toward more mature lamellar bone with increased structural organization, suggesting a transition to the remodeling phase (Figure 5). Low-magnification views (Figure 5(A.1–D.1)) revealed a more mature hard tissue architecture, with bone consistently occupying the healing chambers and closely surrounding the implant structures. High-magnification images (Figure 5(A.2–D.2)) confirmed the presence of active bone remodeling sites, as well as increased bone apposition to the implant surface across all groups.

**Figure 6:**
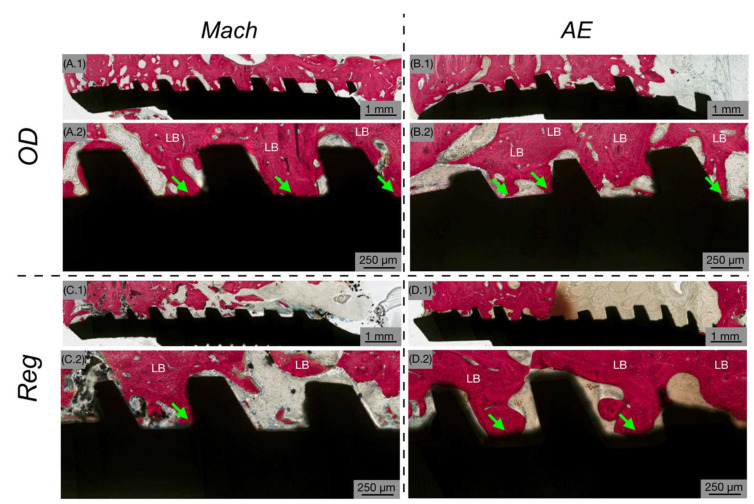
Histological analysis at 24 weeks showing Mach (**A**,**C**) and AE (**B**,**D**) implants with OD (**A**,**B**) and Reg (**C**,**D**) drilling techniques. The implant is shown in black, while bone is shown in red. Low magnification (**A.1**–**D.1**) illustrates dense, mature bone bridging in the healing chambers around the implant. High magnification (**A.2**–**D.2**) confirms the presence of dense, highly organized lamellar bone (LB) and bone in direct apposition to the implant surface (green arrows).

At 24 weeks, the peri-implant bone exhibited dense, well-organized lamellar architecture across all groups (Figure 6). Low-magnification images (Figure 6(A.1–D.1)) showed dense, mature bone bridging the healing chambers and fully encompassing the implant structures. High-magnification views (Figure 6(A.2–D.2)) confirmed the presence of highly organized lamellar bone, and bone in direct contact with the implant surface, reflecting advanced stages of osseointegration.

**Figure 7:**
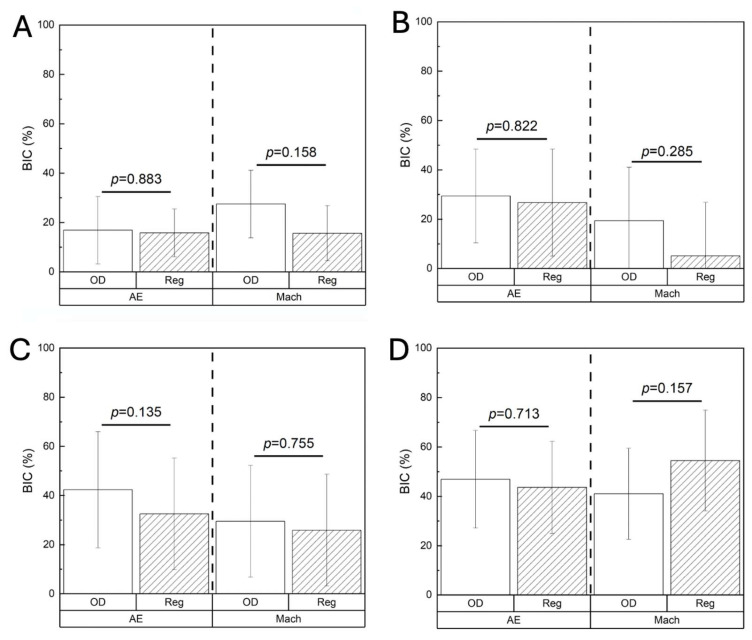
BIC (%) at (**A**) 3 weeks, (**B**) 6 weeks, (**C**) 12 weeks, and (**D**) 24 weeks. Data presented as mean ± 95% CI. *p* < 0.05 is statistically significant.

**Figure 8:**
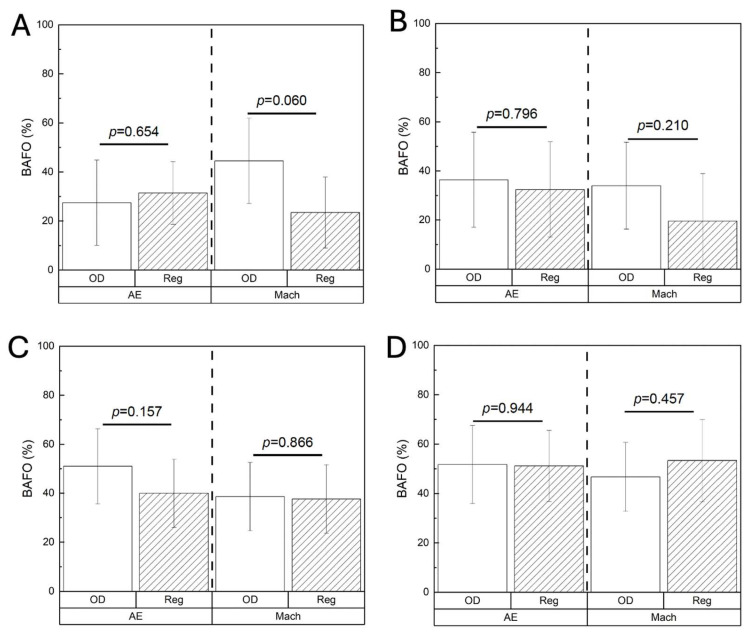
BAFO (%) at (**A**) 3 weeks, (**B**) 6 weeks, (**C**) 12 weeks, and (**D**) 24 weeks. Data presented as mean ± 95% CI. *p* < 0.05 is statistically significant.

BIC at 3 weeks was statistically homogenous between the OD and Reg drilling protocols, regardless of the implant surface (AE: *p* = 0.883; Mach: *p* = 0.158) (Figure 7A). This pattern remained consistent at 6 weeks (AE: *p* = 0.822; Mach: *p* = 0.285) (Figure 7B), 12 weeks (AE: *p* = 0.135; Mach: *p* = 0.755) (Figure 7C), and 24 weeks (AE: *p* = 0.713; Mach: *p* = 0.157) (Figure 7D). Similar findings were revealed with BAFO irrespective of the implant surface, with no significant differences between OD and Reg drilling at any of the time points evaluated: 3 weeks (AE: *p* = 0.654, Mach: *p* = 0.060) (Figure 8A); 6 weeks (AE: *p* = 0.796, Mach: *p* = 0.210) (Figure 8B); 12 weeks (AE: *p* = 0.157, Mach: *p* = 0.866) (Figure 8C); and 24 weeks (AE: *p* = 0.944, Mach: *p* = 0.457) (Figure 8D).

## 4. Discussion

Surgical drilling is critical for achieving primary implant stability. Prior studies have demonstrated that the OD protocol offers an advantage in low-density bone by compacting the surrounding matrix, thereby enhancing primary stability and promoting subsequent bone remodeling [11,37,38]. Previous studies have also demonstrated that OD can enhance insertion torque, BIC, and early bone healing in low-density bone environments. However, its effectiveness in cortical bone remains inconclusive. For instance, Mercier et al. reported increased insertion torque and bone density using OD in the human mandible [18], whereas other investigations using dense bone models reported little to no advantage over conventional drilling techniques [20,21]. The present study expands upon this body of literature by assessing the effects of OD versus Reg drilling in conjunction with different implant surface textures (AE and Mach) on implant stability in dense mandibular bone. At all evaluated time points and across both surface conditions, OD and Reg drilling protocols produced comparable mechanical and histomorphometric outcomes. In the context of high baseline bone density, the compaction effects of OD may have a limited impact on altering the implant’s mechanical environment.

The insertion torque values in this study were comparable between OD and Reg drilling protocols in osteotomies with diameters comparable to implant diameters regardless of implant surface topography. These findings likely reflect the inherently high frictional resistance during implant placement into dense cortical bone [39]. Although OD has been proposed to enhance insertion torque by compacting the osteotomy trabecular walls, this effect may be reduced in dense bone where trabecular space is limited. Importantly, insertion torque has been shown to be closely tied to bone density. Gilmer and Lang highlighted the strong positive correlation between bone density, insertion torque, and screw pull-out force [40]. These findings highlight that bone density may be a main determinant of primary stability and further explain why OD may have a diminished impact in dense cortical bone [40]. On the other hand, studies by Mercier et al. and Vaddamanu et al. have reported increased torque and bone formation associated with OD; however, the inclusion of multiple anatomical sites with varying densities in those investigations may account for the observed differences [18,19]. Notably, in clinical practice, not all mandibular regions are composed of dense cortical bone, particularly in aging populations at risk for osteopenia. 

Histomorphometric analysis showed no statistically significant differences in BIC or BAFO between groups. These results align with findings from a recent clinical trial, which similarly reported no significant differences in implant stability or success rates between OD and conventional drilling in dense bone [21]. Although OD tended to yield numerically higher average values of BIC and BAFO, particularly during the early healing period (3-6 weeks), these differences were not statistically significant. These trends align with previous findings suggesting that OD may enhance early healing kinetics [31,41]. However, the effect size may not have been sufficient to reach significant differences in dense cortical environments and could be explored in future studies.

Implant surface treatment did not influence outcome variables in this study. While surface modifications have been widely reported to enhance osseointegration in low-density bone, particularly when combined with the OD technique [31], their effect may be less pronounced in dense bone. OD enhances mechanical interlocking at the bone–implant interface, whereas the acid-etched surfaces facilitate cellular recruitment and migration from the surrounding bone marrow towards the implant surface [42]. The minimal differences observed between AE and Mach surfaces in the present study suggest that the characteristics of high-density bone may mitigate the influence of surface roughness during the early phases of osseointegration. These findings are consistent with those of Jinno et al., who reported no significant differences when evaluating the effects of AE surface treatment alone in cortical bone [42]. This may be explained by the low vascularity, slower bone turnover, and reduced cellular activity in cortical bone. Thus, implant stability in cortical bone may be more reliant on implant macrogeometry, and as such, the effect of OD may be dependent upon the bone chamber design of the implants in this setting [42]. 

Intriguingly, several studies have reported improved implant performance in dense cortical bone when combining AE with grit blasting [29,30,32,33,34,35]. This combination of techniques creates larger surface irregularities, in addition to the influence of bone chamber design for cortical bone, increasing the mechanical interaction potential between the implant and bone. When this is followed by AE, microscale roughness is superimposed onto the macro-texture, producing a hierarchically structured surface that is both mechanically and biologically favorable. Future studies should aim to assess this synergy between macro- and micro-roughness and potential modulatory effects on OD in improving long-term osseointegration.

## 5. Conclusions

Histological analysis revealed excellent osseointegration across all groups, irrespective of drilling method, in high-density bone settings. Taken together, these findings suggest that the success of implants with designated bone chambers in cortical bone may be less dependent on drilling protocol, or implant surface treatment relative to trabecular bone. With the OD technique, no negative outcomes such as necrosis, inflammation, or microfractures were observed. It is critical to underscore that the osteotomy diameter in cortical bone must closely match the implant’s major diameter, and undersizing should be avoided when using OD techniques. In clinical practice, both drilling techniques remain viable options for implant placement in cortical bone regions, and either technique may be appropriate depending on surgeon preference or clinical context.

## Data Availability

The raw data supporting the conclusions of this article will be made available by the authors on request.
